# Novel anticoagulants versus vitamin K antagonists for cardioversion of non- valvular atrial fibrillation – a meta-analysis of more than 17000 patients

**DOI:** 10.1038/s41598-019-39925-5

**Published:** 2019-02-28

**Authors:** Raluca Ileana Mincu, Amir Abbas Mahabadi, Matthias Totzeck, Tienush Rassaf

**Affiliations:** West German Heart and Vascular Center, Department of Cardiology and Vascular Medicine, University Hospital Essen, Essen, Germany

## Abstract

Non-vitamin K antagonist oral anticoagulants (NOACs) have proven a favorable risk-benefit profile compared to vitamin K antagonists (VKAs) for preventing stroke and systemic embolism in patients with non-valvular atrial fibrillation (AF), but actual data are not sufficiently powered to extend this profile on patients with AF that undergo cardioversion. We aimed to compare outcomes after cardioversion of AF under NOACs vs. VKAs. We systematically searched Pubmed, Cochrane, SCOPUS, and Web of Science databases for studies published until October 2017. A total of 17506 patients from 11 studies were included. Treatment with NOACs was associated with similar relative risks (RR) of stroke and systemic embolism, hemorrhagic stroke, myocardial infarction, cardiovascular death, and all cause death compared to VKAs treatment. The RR of ischemic stroke was lower in the NOACs group. The risk of major bleeding was similar across treatment groups. Treatment with NOACs in patients with non-valvular AF that undergo cardioversion seems to be as safe and effective as the use of classical VKAs, with a better profile for ischemic stroke. Clinical Trial Registration: PROSPERO Registry, CRD42018086181 https://www.crd.york.ac.uk/prospero/display_record.php?RecordID = 86181.

## Introduction

Although the last years have brought substantial progress in the management of atrial fibrillation (AF), this arrhythmia continues to be one of the major causes of stroke, heart failure, sudden death, and cardiovascular morbidity in the world^1^. The thromboembolic complications following cardioversion of non-valvular AF are more frequent in patients not treated with anticoagulants before the procedure, with the highest rates for patients with heart failure, diabetes mellitus or AF duration more than 12 hours^2,3^. The anticoagulant treatment with classical vitamin K antagonists (VKAs) or the non-vitamin K antagonist oral anticoagulants (NOACs), like the direct thrombin inhibitor dabigatran^4^ and the factor Xa inhibitors apixaban^5^, edoxaban^6^, and rivaroxaban^7^ should start immediately for patients with AF scheduled for cardioversion, in order to reduce these adverse events^1,8^. Although the NOACs would be easier to manage for this group of patients, the actual data is not sufficiently powered to favor NOACs over VKAs in the setting of cardioversion, as it is for the general treatment of patients with AF and indication for anticoagulation^1^. To date, three secondary post-hoc analyses of the RE-LY trial^9^, ARISTOTLE trial^10^, and ROCKET-AF trial^11^ suggested that NOACs show low or comparable rates of thromboembolic events and hemorrhagic complications in patients with AF that undergo cardioversion compared to VKAs. The X-VeRT trial^12^, the first prospective trial for the use of NOACs in the setting of cardioversion, showed once again comparable safety and efficacy compared to VKAs. In a recent post-hoc analysis of the ENGAGE TIMI 48 trial^13^, thromboembolic and major bleeding events in the 30 days post-cardioversion were infrequent (<2%) and similar between edoxaban and warfarin. The prospective randomized, open-label ENSURE-AF trial^14^ concluded that edoxaban could be an effective and safe alternative to the best possible conventional treatment with enoxaparin and VKAs strategy.

The EMANATE study^15^, comparing NOACs to VKAs in anticoagulation naïve patients that underwent cardioversion, suggested, with the limitations of an underpowered study, that the use of apixaban lowers the risk of stroke compared with warfarin, with similar rates of bleeding across the groups. In addition to data from randomized controlled trials (RCT), data from clinical registries, suggested that NOACs appear safe and effective compared to warfarin, with low rates of thromboembolic and bleeding complications^16–19^. However, the actual data is not sufficiently powered to firmly recommend NOACs in patients with AF that undergo cardioversion. Some meta-analyses have tried to overcome this limitation^20,21^ and reported similar rates of thromboembolism and bleeding between NOACs and VKAs. The recent publication of new data that would substantially increase number of patients analyzed in each of the two groups and the need of describing the entire panel of complications following cardioversion make further research meaningful. We performed a meta-analysis of the studies that compared outcomes of patients with non-valvular AF undergoing cardioversion treated with NOACs with patients treated with VKAs, aiming to (I) enrich statistical power for the evaluation of a potential non-inferiority of NOACs over VKAs, (II) compare data from RCT with large registry studies and (III) achieve sufficient sample size for evaluation of secondary outcome variables.

## Results

### Study selection

A total number of 11 studies met the inclusion criteria^9–19^, with a total of 17506 patients with AF that underwent cardioversion, 7381 in the NOACs group and 10125 in the VKAs group. Of these, 7 studies were randomized trials or post-hoc analysis of a randomized trial and included 8587 patients^9–15^, whereas 4 were cohort studies with 8927 patients were included^16–19^.

**Figure 1:**
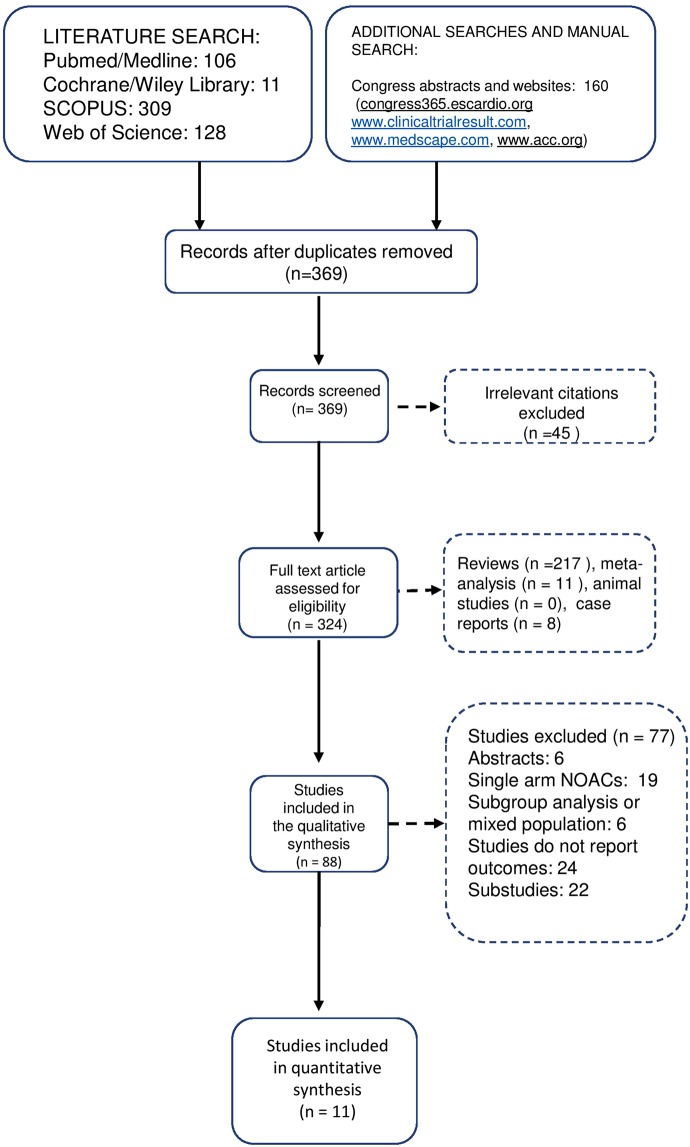
PRISMA selection chart.

The study selection process is depicted in Fig. 1 as a PRISMA flowchart. The characteristics of the included studies are detailed in Supplementary Table 1. The mean follow-up time of the included studies varied between 30 days and 6 months. The selected studies had a high quality, according to the quality criteria of the Cochrane Handbook^22^. (Supplementary Fig. 1).

### Stroke and systemic embolism

**Figure 2:**
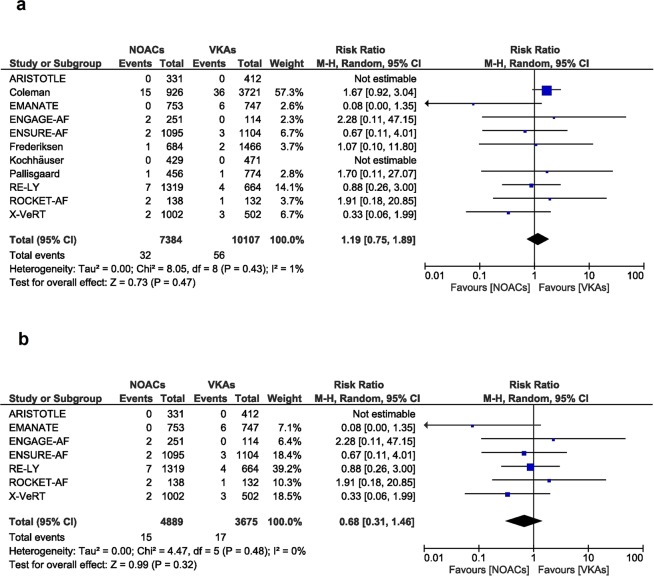
Overall and individual study estimates of the RR of stroke and systemic embolism associated with NOACs treatment vs. VKAs treatment in all studies (**a**) and in the randomized controlled studies (**b**). Parallelogram boxes denote the RR, and horizontal lines represent 95% confidence intervals. RR = Risk Ratio, NOACs = non-vitamin K antagonist oral anticoagulants, VKAs = vitamin K antagonists.

No difference in stroke or systemic embolism was observed (RR [95% CI] = 1.19 [0.75, 1.89], p = 0.47, Fig. 2a). A total of 17491 patients, 7384 in the NOACs group and 10107 in the VKAs group from all the 11 studies were included in the meta-analysis^9–19^, No relevant heterogeneity and inconsistency between groups was present, as reflected by the I^2^ of 1% and p value of 0.43 (Fig. 2a) and by the funnel plot (Supplementary Fig. 2).

Limiting the analysis to the 7 randomized trials^9–15^, result remained unchanged (RR [95% CI] = 0.68 [0.31, 1.46], p = 0.32). Likewise, no important heterogeneity and inconsistency was reflected by the I^2^ of 0% and a p value of 0.48 (Fig. 2b).

### Ischemic stroke and hemorrhagic stroke

**Figure 3:**
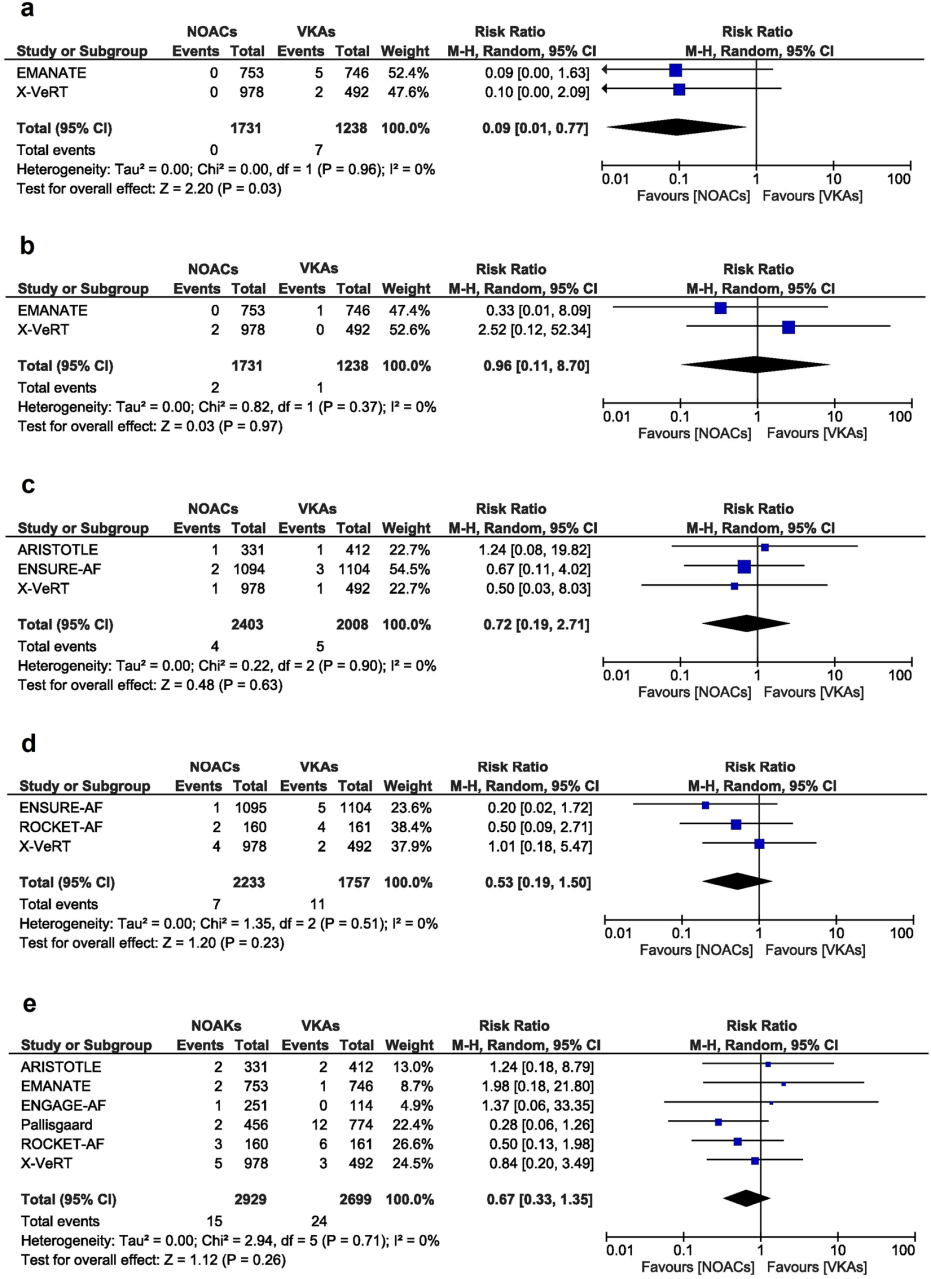
Overall and individual study estimates of the RR of ischemic stroke (**a**), hemorrhagic stroke (**b**), myocardial infarction (**c**), cardiovascular death (**d**) and all cause death (**e**) associated with NOACs treatment vs. VKAs treatment. Parallelogram boxes denote the RR, and horizontal lines represent 95% confidence intervals. RR = Risk Ratio, NOACs = non-vitamin K antagonist oral anticoagulants, VKAs = vitamin K antagonists.

The EMANATE and X-VeRT trials reported the incidences of ischemic stroke^12,15^. Risk of ischemic stroke was significantly lower in the NOACs group compared to the VKAs group (RR [95% CI] = 0.09 [0.01, 0.77], p = 0.03, Fig. 3a). The heterogeneity and inconsistency between the studies was very low, as reflected by the I^2^ of 0% and a p value of 0.96.

The relative risk of hemorrhagic stroke was similar between the two groups of patients that underwent cardioversion for atrial fibrillation, as reflected by a RR of 0.96 (95% CI [0.11, 8.70], p = 0.97). The analysis included two studies^12,15^, with 2970 patients that underwent cardioversion, 1731 in the NOACs group and 1238 in the VKA group. The heterogeneity and inconsistency between the studies was very low, as reflected by the I^2^ of 0% and a p value of 0.37 (Fig. 3b).

### Myocardial infarction

For analysis of myocardial infarction, data from 3 available studies^10,12,14^ was pooled, which included 4411 patients (2403 in the NOACs group and 2008 in the VKAs group). The risk of myocardial infarction was similar between the two groups of patients (RR [95% CI] = 0.72 [0.19, 2.71], p = 0.63). The heterogeneity and inconsistency between the studies was not significant, as reflected by the I^2^ of 0% and a p value of 0.9 (Fig. 3c).

### CV and all-cause death

Data on CV death was reported in 3 studies with overall 3990 patients (2233 patients in the NOACs group and 1757 patients in the VKAs group)^11,12,14^. No relevant difference in RR for CV death for patients with AF that underwent cardioversion was observed comparing patients treated with NOACs compared to VKAs (RR [95% CI] = 0.53 [0.19, 1.50], p = 0.23). The heterogeneity and inconsistency between groups was not significant, with I^2^ of 0% and a p value of 0.51 (Fig. 3d).

Likewise, no relevant difference in all-cause death in the NOACs group comparted to the VKAs group was observed (RR [95% CI] = 0.67 [0.33, 1.35], p = 0.26) when including 5629 patients from 6 studies (2929 patients in the NOACs group and 2699 patients in the VKAs group)^10–13,15,18^. The heterogeneity and inconsistency between groups was not significant, with I^2^ of 0% and a p value of 0.71 (Fig. 3e).

### Major bleeding

**Figure 4:**
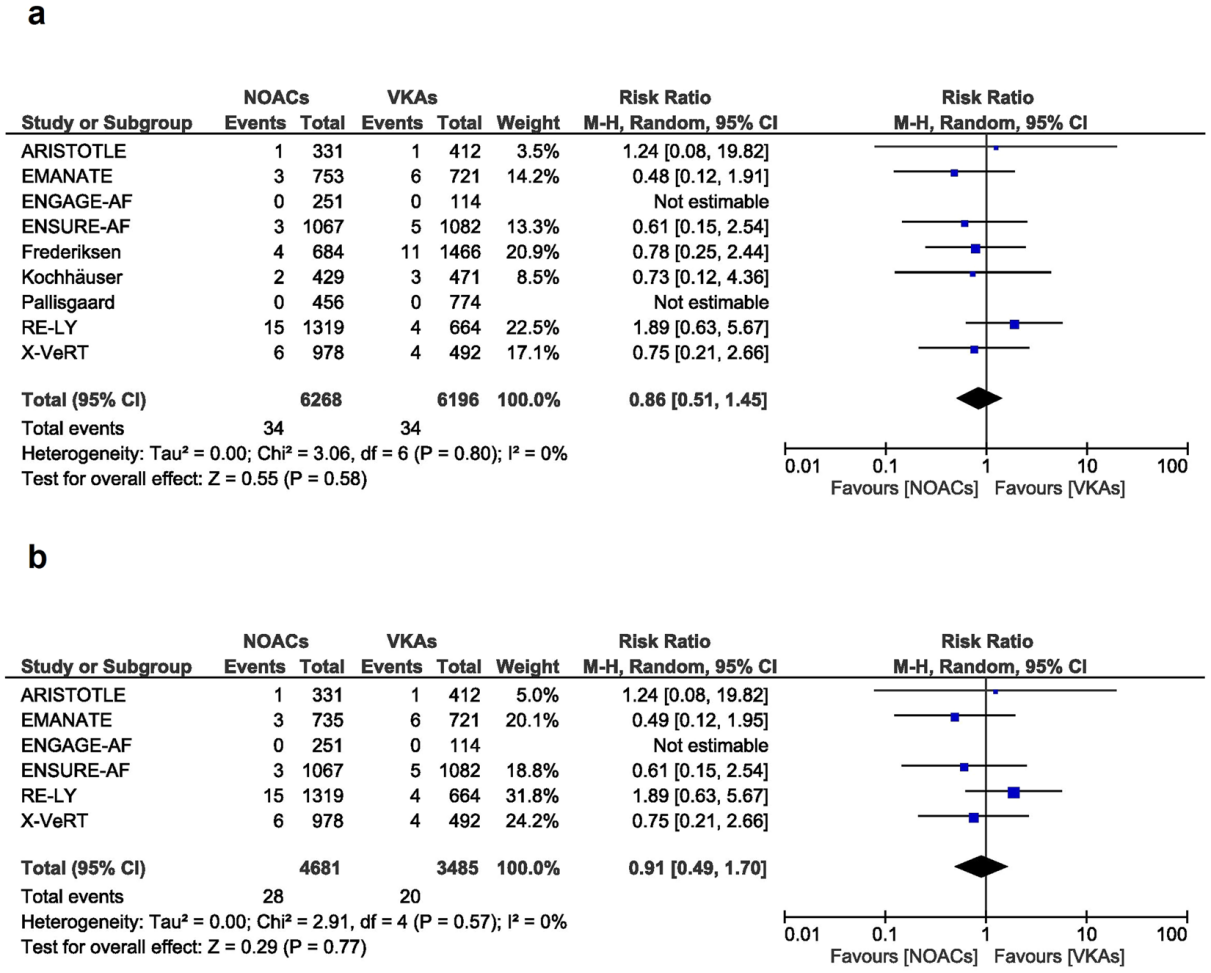
Overall and individual study estimates of the RR of major bleeding associated with NOACs treatment vs. VKAs treatment in all studies (**a**) and in the randomized controlled studies (**b**). Parallelogram boxes denote the RR, and horizontal lines represent 95% confidence intervals. RR = Risk Ratio, NOACs = non-vitamin K antagonist oral anticoagulants, VKAs = vitamin K antagonists.

The risk of major bleeding was similar between the patients treated with NOACs and the patients treated with VKAs (RR [95% CI] = 0.86 [0.51, 1.45], p = 0.58). The analysis included 6268 in the NOACs group and 6196 in the VKAs group. The heterogeneity and inconsistency between groups was not important, as reflected by the I^2^ of 0% and a p value of 0.80 (Fig. 4a).

These results did not change when excluding the 3 cohort studies^17–19^. In this case we observed a similar major bleeding risk between the groups (RR [95% CI] = 0.91 [0.49, 1.7], p = 0.77) and no important heterogeneity and inconsistency (I^2^: 0%, p-value: 0.57, Fig. 4b).

The subtypes of bleeding events cannot be analyzed separately, as most of the studies included in this analysis report them as a composite of all major bleeding events, defined according to the International Society of Thrombosis and Hemostasis definition from 2005.

### Subgroup analysis

We performed a subgroup analysis regarding differences in duration of follow-up duration, and divided the studies in a 30 days follow-up subgroup with 6 studies^9–13,15^, and a more than 30 days follow-up subgroup with 5 studies^14,16–19^. The impact of NOACs as compared to VKAs therapy on stroke and systemic embolism rates were similar in both subgroups, showing that the differences in duration of follow-up had no influence on the main outcome. (Supplementary Fig. 3).

## Discussion

In the present meta-analysis, including over 17000 patients with non-valvular AF that underwent cardioversion, we observed no differences regarding stroke or systemic embolism, hemorrhagic stroke, myocardial infarction, CV or all-cause death, or major bleeding risk when comparing NOACs to VKAs therapy. Moreover, the relative risk of ischemic stroke was lower for the patients treated with NOACs. Data was consistent for RCT as well as for the registry data. According to our analysis, the use of NOACs for patients with non-valvular AF that undergo cardioversion seems to be as safe and effective as the use of VKAs, with the advantage of a lower relative risk of ischemic stroke.

This study analyses all the evidences available at this point regarding the outcomes of patients with non-valvular AF that underwent cardioversion treated with NOACs or VKAs, and brings additional data regarding the spectrum of the adverse events mentioned in different studies. Other available evidence^20,21,23,24^ included a significant smaller number of patients, and provided mainly data about stroke and major bleeding, while results were concordat with our findings. The study included up-to-date publications, provides details on rates of ischemic and hemorrhagic stroke, myocardial infarction, as well as CV death, and includes not only RCT, but also registry data.

The anticoagulant treatment should start immediately for patients scheduled for cardioversion, in order to reduce the adverse events^8^. A major disadvantage of VKAs in the setting of cardioversion is the delayed onset of action and the necessity of bridging therapy with non-fractionated heparin or low-molecular-weight heparin for the patients with international normalized ratios out of the therapeutic range or for the initiation of the therapy, whereas the NOACs have a peak activity level in 1 to 3 hours and a better bioavailability. Furthermore, the monitoring of the treatment with VKAs necessitates regular measuring of the international normalized ratio, whereas the treatment with NOACs eliminates this inconvenient^1^. For these reasons, and considering the fact that the NOACs seem to be at least as effective as VKAs in patients with non-valvular AF that undergo cardioversion, the efforts to define NOACs an evidence-based indication for these patients represent a milestone for the clinical practice.

The clinical trials include highly selected patients that do not always reflect the clinical reality. However, we observed consistent results for both RCT and clinical registry data, supporting the safe use of NOACs in patients undergoing cardioversion in daily clinical routine.

The up-to-date evidences regarding the use of NOACs for cardioversion of non-valvular AF derive from post-hoc analyses of the bigger RCT^9–11^ with the major disadvantage of the prolonged time of anticoagulation treatment. In the RE-LY study^9^ the comparison of dabigatran 150 mg twice a day to warfarin showed similarly low rates of stroke (0.3% vs. 0.6%, p = 0.4048) and major bleeding (0.6% vs. 0.6%, p = 0.9865) at 30 days after cardioversion. Similarly, the ARISTOTLE trial showed comparable low rates of stroke and systemic embolism (0% vs. 0%) and major bleeding between apixaban and warfarin at 30 day after cardioversion (0.35% vs 0.3%)^10^. The incidence of stroke and systemic embolism was similar for the patients treated with rivaroxaban vs. warfarin (1.61% vs. 2.48%) in the ROCKET-AF study. The higher rate of events compared to other studies derives from the design of the study that included patients with more comorbidities. Furthermore, the incidence of major bleeding in the rivaroxaban vs. warfarin group was high (19.35% vs. 14.05%) in ROCKET-AF study^11^. Rivaroxaban was further compared to warfarin in X-VeRT, an open label RCT phase IIIb in the setting of cardioversion, and showed similarly low rates of stroke (0.2% vs. 0.41%) and major bleeding (0.61% vs. 0.80%)^12^. In a recent post-hoc analysis of the ENGAGE TIMI 48 trial^13^, thromboembolic and major bleeding events in the 30 days post-cardioversion were infrequent (<2%) and similar between edoxaban and warfarin. The prospective randomized, open-label ENSURE-AF trial^14^ that compared edoxaban to warfarin, showed low rates of stroke, myocardial infarction or cardiovascular death or major bleeding (<1%). The most recent RCT, the EMANATE trial^15^, shows similar rates of events between apixaban and warfarin.

The data derived from cohort studies show the same low equivalent rates of thromboembolic and bleeding complications between NOACs and VKAs, with the advantage of being more related to clinical reality and the disadvantages of the lack of randomisation^16–19^. Our meta-analysis reports the same low (<1%) and equivalent rate of adverse events between NOACs and VKAs, except for the ischemic stroke, where the NOACs showed superiority, while the conclusions derived from every study taken separately are not sufficient to impose the routine use of NOACs for patients with non-valvular AF that undergo cardioversion.

### Limitations

This meta-analysis has some limitations that need to be mentioned. Firstly, the analysis included both RCT and non-RCT data, which could affect the overall quality of the data, but the real word data provided from cohort studies gives us a better insight into the clinical reality and ultimately confirmed results derived from RCT. Secondly, due to the lack of detailed information reported in the included studies, we were not able to perform subgroup analyses regarding the dose of NOACs, the therapeutic level of anticoagulation around the time of cardioversion, the time in therapeutic range for VKAs or the time between NOACs administration and cardioversion. Thirdly, the inclusion criteria and therefore also baseline characteristics differed between included trials, which can create differences in outcomes. Moreover, the original studies had different designs, and the post-hoc analyses included cannot be considered RCT. Additionally, the ROCKET-AF trial also included patients that underwent ablation for cardioversion. All these elements could additionally increase the risk of bias of this meta-analysis. Fourthly, in some trials, investigators had the possibility to replace the blinded study medication temporarily with open-label VKAs for the cardioversion period and this may interfere with the analysis of outcomes. Fifthly, one study^9^ reported the outcomes related to the number of cardioversions and not the number of patients that underwent cardioversion.

## Conclusion

Treatment with NOACs in patients with non-valvular AF that undergo cardioversion seems to be as save and effective as the use of classical VKAs, with a better profile for ischemic stroke. These results encourage the use of NOACs in the setting of AF that necessitates cardioversion.

## Methods

This meta-analysis was performed in accordance with the Preferred Reporting of Items for Systematic Meta-Analysis (PRISMA) reporting guidelines (Supplementary Table 2) and followed the Cochrane Handbook for Systematic Reviews of Interventions recommendations^22,25^. The methodology used in this meta-analysis has been already published^26,27^. The study was registered in PROSPERO Registry (CRD42018086181).

### Sources of information and search strategies

A systematic search was conducted using the Pubmed, Cochrane, SCOPUS, and Web of Science databases; the major cardiology websites (congress365.escardio.org, www.clinicaltrialresult.com, www.medscape.com, and www.cardiosource.com); and the abstracts or presentations from annual meetings of the major cardiovascular societies to identify relevant studies published until 1^st^ of November 2017, exclusively. We made our search specific and sensitive using MeSH (Medical Subject Headings) terms and free text and considered studies published in any language. Search terms used were “atrial fibrillation”, “cardioversion”, “apixaban”, “dabigatran”, “edoxaban”, “rivaroxaban”, “warfarin”, “vitamin K antagonists”.

The following inclusion criteria were used:

1. Studies involving patients with non-valvular AF that underwent cardioversion treated with one of the approved NOACs, including apixaban, rivaroxaban, edoxaban, and dabigatran, versus VKAs.
2. Assignment of patients to at least two treatment groups: NOACs group and VKAs group.
3. Minimum 30-day follow-up period.

The following exclusion criteria were used:

1. Review studies, animal and *in vitro* studies, meta-analyses, and case-reports.
2. Single-arm treatment studies, or treatment with NOACs in both groups.
3. Studies not reporting on the selected outcomes.
4. Subgroup population studies (elderly population, population from a certain geographic region).

After removing duplicates, RIM, AAM and MT reviewed the abstracts independently. The discrepancies in results were discussed with TR. The complete publication was reviewed, when the inclusion criteria appeared to be fulfilled. All the investigators reviewed the full texts of the selected studies.

### Data extraction and quality assessment

Two authors (RIM and AAM) performed the data extraction independently. The following information was gathered: publication details (name of the first author and year of publication); study design; characteristics of the study population (sample size, age and gender distribution); dose of NOACs and VKA; median follow-up; and selected study endpoints.

The quality of the included studies was assessed according to the Cochrane Risk of Bias Tool. Each study was assessed separately for the following biases: (A) random sequence generation (selection bias); (B) allocation concealment (selection bias); (C) blinding of participants and personnel (performance bias); (D) blinding of outcome assessment (detection bias); (E) incomplete outcome data; (F) selective reporting (reporting bias) and (G) other bias (measurement error, observer variability, dose of drug, length of follow-up, and characteristics of participants)^22^.

### Study endpoints

The study endpoints were: stroke or systemic embolism, hemorrhagic stroke, ischemic stroke, myocardial infarction, major bleeding, cardiovascular death and all cause death. Stroke was defined as the abrupt onset of a nontraumatic, focal neurological deficit lasting at least 24 h, or systemic embolism, defined as symptoms consistent with acute loss of blood to a noncerebral artery confirmed by autopsy, angiography, vascular imaging, or some other objective testing^28^. A diagnosis of primary hemorrhagic stroke requires documentation by imaging of hemorrhage in the cerebral parenchyma or in the subdural or subarachnoid space or evidence of hemorrhage obtained by lumbar puncture neurosurgery or identified at autopsy^29^. Myocardial infarction (MI) was defined as symptoms with biomarker elevation at least 2 times greater than normal (creatine kinase, creatine kinase-myocardial band, or troponin) or with new Q waves in ≥2 contiguous leads. Death was classified as cardiovascular (stroke, systemic embolism, MI, sudden death, heart failure, or indeterminate) or noncardiovascular^30^. The primary safety outcome was major bleeding as defined by the International Society of Thrombosis and Haemostasis for all RCT and most cohort trials^31^. Only in the study by Pallisgaard *et al*., major bleeding was defined according to the ICD 10 codes^18^ and in the study by Kochhäuser *et al*., major bleeding was defined as clinically important if it required hospitalization, transfusion, or cessation of anticoagulation for >7 days^17^.

### Statistical analysis

The statistical analysis protocol has been published before^26^. The meta-analysis was conducted on eligible studies by dividing the patients into the following two groups: the NOACs group, which included patients with atrial fibrillation that underwent cardioversion treated with NOACs and the VKA group, which included patients with atrial fibrillation that underwent cardioversion treated with VKA. The number of patients with adverse events receiving NOACs was compared to that of the VKAs group in the same study. The data are expressed as the risk ratios (RR) and 95% confidence intervals (95% CI)^32^. For the analysis, we used mainly the random-effects models, because this model weights the studies relatively more equally than a fixed-effects model analysis. Heterogeneity between studies was assessed using the Q statistic, and inconsistencies were quantified using the I^2^ statistic. We considered the presence of significant heterogeneity at the 10% level of significance. A value of I^2^ of 0% to 40% denotes that heterogeneity might not be important, 30% to 60% may represent moderate heterogeneity, 50% to 90% may represent substantial heterogeneity and 75% to 100% represents considerable heterogeneity^22^. The presence of publication bias was assessed using the funnel plot test (Egger’s test). Studies with high precision are plotted near the average, and studies with low precision are spread evenly on both sides of the average, creating a roughly funnel-shaped distribution. Deviation from this shape indicates publication bias^33^.The funnel plot test was not used when the analysis included fewer than 10 studies^22^. A sensitivity analysis was performed by excluding each study in turn from the analysis to determine the relative importance of each study, as well as subgroup analysis that excluded the cohort studies. The analyses were conducted using Review Manager version 5.3 (Revman, The Cochrane Collaboration, Oxford, United Kingdom).

## Supplementary information

Supplementary material
