# Supplementary material for: Novel anticoagulants versus vitamin K antagonists for cardioversion of non- valvular atrial fibrillation – a meta-analysis of more than 17000 patients
Source: Sci Rep. 2019 Feb 28;9:3011. doi: 10.1038/s41598-019-39925-5 (PMC6395612; doi:10.1038/s41598-019-39925-5)
Supplement: Supplementary file 1 — Supplementary material [file 41598_2019_39925_MOESM1_ESM.pdf]

Novel anticoagulants versus vitamin K antagonists for cardioversion of non- valvular atrial fibrillation – a meta-analysis of more than 17000 patients

Supplementary Material

Raluca I. Mincu, PhD[1], Amir A. Mahabadi, MD[1], Matthias Totzeck, MD[1], Tienush Rassaf, MD[1*]

[1]West German Heart and Vascular Center, Department of Cardiology and Vascular Medicine, University Hospital Essen, Essen, Germany

[*]Corresponding author:

Tienush Rassaf, MD

West German Heart and Vascular Center

Department for Cardiology and Vascular Medicine

University Hospital Essen

Hufelandstr. 55, 45147 Essen, Germany

Tel.: +49 201 723 4801

Fax: +49 201 723 5401

E-mail: Tienush.Rassaf@uk-essen.de

Supplementary Table 1. Characteristics of the included studies

Study	Acronim of the study	Year of publication	Study type	Sample size	Study drug	Number of patients or cardioversions (CV) in each arm	Median age	% males	Median follow-up
Nagarakanti et al	RE-LY	2011	Post-hoc analysis of an open label RCT	1270	Dabigratran 110 mg	647 CV	NR	NR	30 days
					Dabigratan 150 mg	672 CV	NR	NR	
					Warfarin (target INR = 2.5)	664 CV	NR	NR	
Piccini et al	ROCKET-AF	2013	Post-hoc analysis of a double blind RCT	321	Rivaroxaban 20 mg or 15 mg	160	68	66	30 days
					warfarin (target INR = 2.5)	161	71	59	
Flaker et al	ARISTOTLE	2014	Post-hoc analysis of a double blind RCT	540	Apixaban 5 mg or 2.5 mg	265	67	73	30 days
					warfarin (target INR = 2.5)	275	67	72	
Capatto et al	X-VeRT	2014	Open label RCT IIIb	1504	Rivaroxaban 20 mg or 15 mg	978	64	72	30 days
					warfarin or another VKA (target INR = 2.5)	492	64	73	
Plitt et al	ENGAGE-AF	2016	RCT Double	365	Edoxaban	251	70	NR	30 days

	TIMI 48		blind, double dummy		60/30 mg or Edoxaban 30/15 mg)				
					Warfarin (target INR = 2.5)	114	72	NR	
Goette et al	ENSURE	2016	multicentre, prospective, randomised, open-label, blinded-endpoint	2199	edoxaban 60 mg or 30 mg	1095	64	66	58 days
					warfarin (target INR = 2-3)	1104	64	65	
Ezekowitz et al	EMANATE	2017	randomized, prospective, open-label	1500	Apixaban 5 mg or 2.5 mg	753	NR	NR	30 days
					warfarin (target INR = 2-3)	747	NR	NR	
Kochhäuser et al	none	2014	Retrospective cohort	900	Dabigatran 150 or 110 mg	288	65	63	6 months
					Rivaroxaban 20 or 15 mg	141	62	63	
					VKAs (target INR = 2-3)	471	68	64	
Pallisgaard et al	none	2015	Retrospective cohort	1230	Dabigatran 150 or 110 mg	456	66	72	28 weeks
					Warfarin (target INR = 2-3)	774	67	73	
Coleman et al	none	2015	Retrospective cohort	4647	Dabigatran 150 or 110 mg	719	64	77	8 weeks
					Rivaroxaban 20 or 15 mg	159	66	77	

					Apixaban 5 or 2.5 mg	48	66	75	
					Warfarin (target INR = 2-3)	3721	67	68	
Frederiksen et al	none	2017	Prospective cohort	2150	NOACs	684	NR	NR	60 days
					Warfarin (target INR = 2-3)	1466	NR	NR	

Supplementary Table 2. PRISMA checklist

Section/topic	#	Checklist item	Reported on page #
**TITLE**			
Title	1	Identify the report as a systematic review, meta-analysis, or both.	1
**ABSTRACT**			
Structured summary	2	Provide a structured summary including, as applicable: background; objectives; data sources; study eligibility criteria, participants, and interventions; study appraisal and synthesis methods; results; limitations; conclusions and implications of key findings; systematic review registration number.	2
**INTRODUCTION**			
Rationale	3	Describe the rationale for the review in the context of what is already known.	3,4
Objectives	4	Provide an explicit statement of questions being addressed with reference to participants, interventions, comparisons, outcomes, and study design (PICOS).	3,4
**METHODS**			
Protocol and registration	5	Indicate if a review protocol exists, if and where it can be accessed (e.g., Web address), and, if available, provide registration information including registration number.	2, 12
Eligibility criteria	6	Specify study characteristics (e.g., PICOS, length of follow-up) and report characteristics (e.g., years considered, language, publication status) used as criteria for eligibility, giving rationale.	12-15
Information sources	7	Describe all information sources (e.g., databases with dates of coverage, contact with study authors to identify additional studies) in the search and date last searched.	12, Figure 1
Search	8	Present full electronic search strategy for at least one database, including any limits used, such that it could be repeated.	12,13
Study selection	9	State the process for selecting studies (i.e., screening, eligibility, included in systematic review, and, if applicable, included in the meta-analysis).	12,13, Figure 1
Data collection process	10	Describe method of data extraction from reports (e.g., piloted forms, independently, in duplicate) and any processes for obtaining and confirming data from investigators.	13,14

Data items	11	List and define all variables for which data were sought (e.g., PICOS, funding sources) and any assumptions and simplifications made.	12-14 Supplementary Table 1
Risk of bias in individual studies	12	Describe methods used for assessing risk of bias of individual studies (including specification of whether this was done at the study or outcome level), and how this information is to be used in any data synthesis.	14-15, Supplementary Figure 1
Summary measures	13	State the principal summary measures (e.g., risk ratio, difference in means).	14, 15, Supplementary Figure 1
Synthesis of results	14	Describe the methods of handling data and combining results of studies, if done, including measures of consistency (e.g., I^2) for each meta-analysis.	14, 15

**Section/topic**	**#**	**Checklist item**	**Reported on page #**
Risk of bias across studies	15	Specify any assessment of risk of bias that may affect the cumulative evidence (e.g., publication bias, selective reporting within studies).	Supplementary Figure 1
Additional analyses	16	Describe methods of additional analyses (e.g., sensitivity or subgroup analyses, meta-regression), if done, indicating which were pre-specified.	14, 15
**RESULTS**			
Study selection	17	Give numbers of studies screened, assessed for eligibility, and included in the review, with reasons for exclusions at each stage, ideally with a flow diagram.	5, Figure 1
Study characteristics	18	For each study, present characteristics for which data were extracted (e.g., study size, PICOS, follow-up period) and provide the citations.	Supplementary Table 1
Risk of bias within studies	19	Present data on risk of bias of each study and, if available, any outcome level assessment (see item 12).	Supplementary Figure 1, Supplementary Figure 2
Results of individual studies	20	For all outcomes considered (benefits or harms), present, for each study: (a) simple summary data for each intervention group (b) effect estimates and confidence intervals, ideally with a forest plot.	5-7, Figures 2-4,

Synthesis of results	21	Present results of each meta-analysis done, including confidence intervals and measures of consistency.	5-7
Risk of bias across studies	22	Present results of any assessment of risk of bias across studies (see Item 15).	Supplementary Figure 1, Supplementary Figure 2
Additional analysis	23	Give results of additional analyses, if done (e.g., sensitivity or subgroup analyses, meta-regression [see Item 16]).	5-7
**DISCUSSION**			
Summary of evidence	24	Summarize the main findings including the strength of evidence for each main outcome; consider their relevance to key groups (e.g., healthcare providers, users, and policy makers).	8
Limitations	25	Discuss limitations at study and outcome level (e.g., risk of bias), and at review-level (e.g., incomplete retrieval of identified research, reporting bias).	10-11
Conclusions	26	Provide a general interpretation of the results in the context of other evidence, and implications for future research.	8-11
**FUNDING**			
Funding	27	Describe sources of funding for the systematic review and other support (e.g., supply of data); role of funders for the systematic review.	18

From: Moher D, Liberati A, Tetzlaff J, Altman DG, The PRISMA Group (2009). Preferred Reporting Items for Systematic Reviews and Meta-Analyses: The PRISMA Statement. PLoS Med 6(6): e1000097. doi:10.1371/journal.pmed1000097

For more information, visit: **www.prisma-statement.org**.

Supplementary Figures

Supplementary Figure 1. The quality of the included studies as analysed per Cochrane Handbook's recommendation.

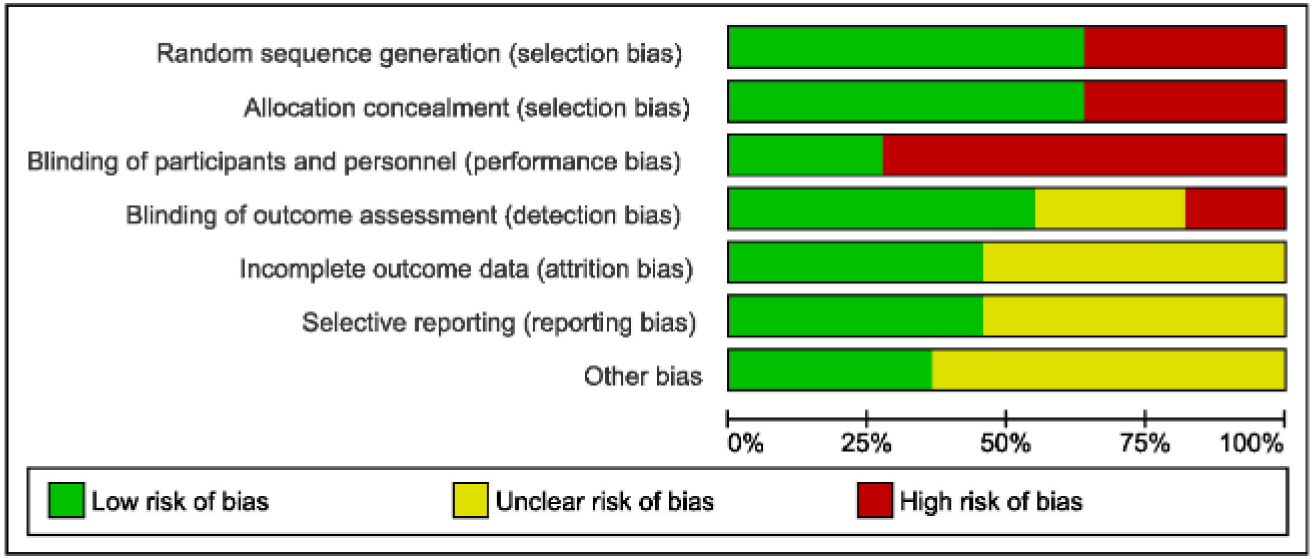

Supplementary Figure 2. Risk of bias for the studies included in the stroke and systemic embolism analysis.

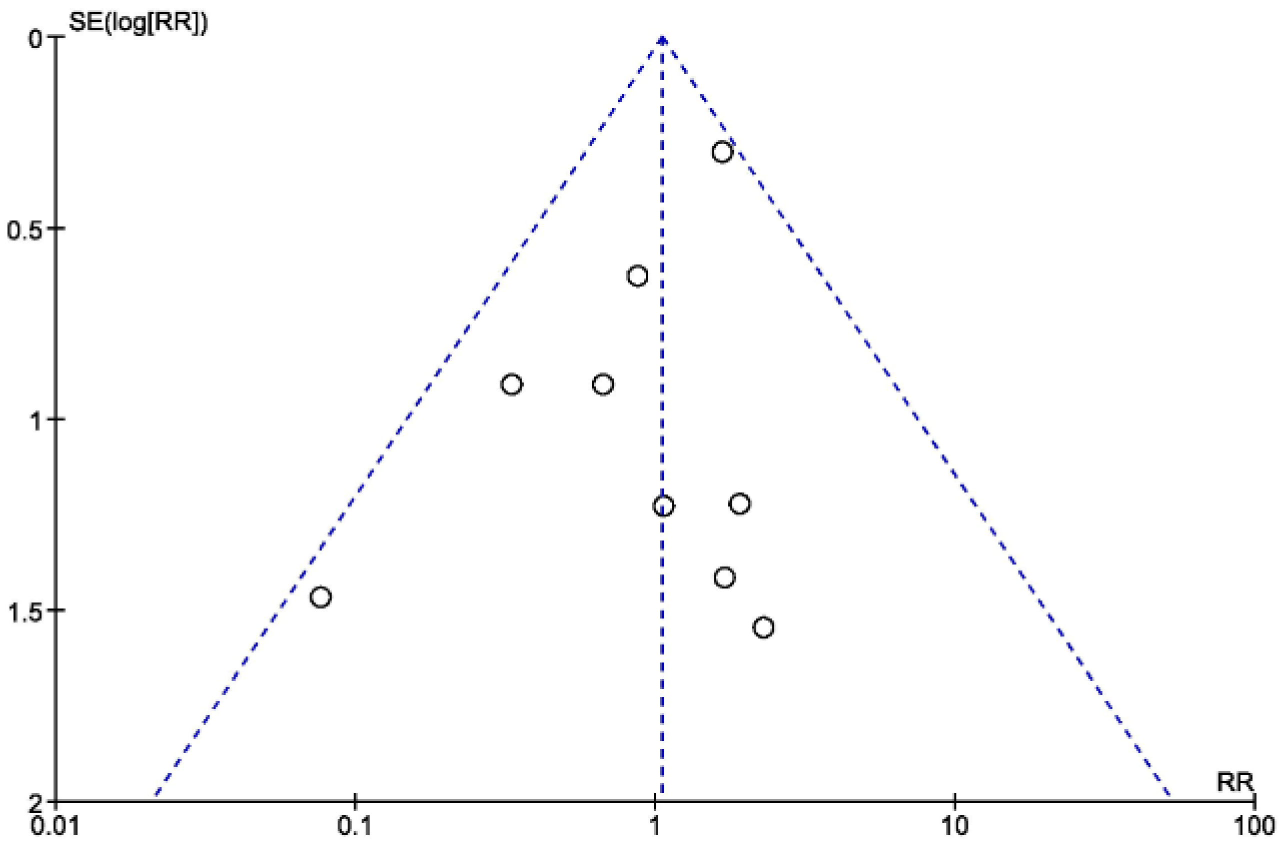

Supplementary Figure 3. Subgroup analysis of RR of stroke and systemic embolism based on the follow-up duration of each study.

	NOACs		VKAs			Risk Ratio
Study or Subgroup	Events	Total	Events	Total	Weight	M-H, Random, 95% CI
Follow-up 30 days	13	3766	14	2571	34.0%	0.63 [0.30, 1.35]
Follow-up more than 30 days	19	3590	42	7560	66.0%	0.95 [0.55, 1.64]
**Total (95% CI)**		**7356**		**10131**	**100.0%**	**0.83 [0.53, 1.29]**
Total events	32		56			

Heterogeneity: Tau² = 0.00; Chi² = 0.74, df = 1 (P = 0.39); I² = 0%

Test for overall effect: Z = 0.83 (P = 0.40)

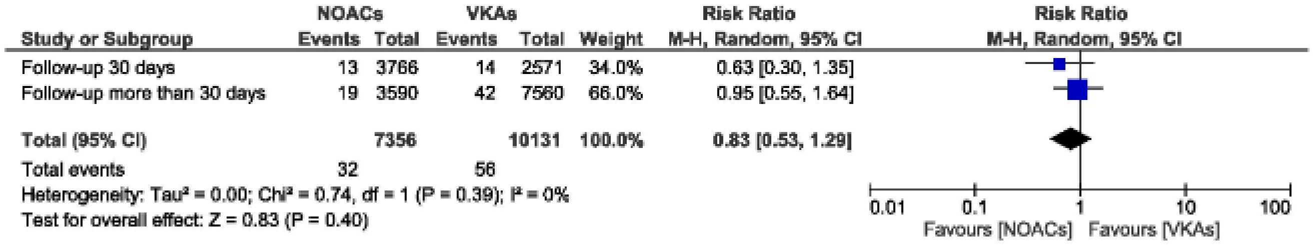

Parallelogram boxes denote the RR, and horizontal lines represent 95% confidence intervals. RR = Risk Ratio, NOACs = non-vitamin K antagonist oral anticoagulants, VKAs = vitamin K antagonists
